# Conference Report on the 2025 Annual Review of the Essential Programme on Immunization in DR Congo: Dealing with Complexity

**DOI:** 10.3390/vaccines14030257

**Published:** 2026-03-11

**Authors:** Audry Mulumba, Franck Mboussou, Pablito Nasaka, Augustin Milabyo Byamwitenga, Aimé Cikomola, Cyril Nogier, Thomas Noel Gaha, Mymy Mwika, Benedict Taa Nguimbis, Bridget Farham, Anne Ancia, Benido Impouma

**Affiliations:** 1 Essential Immunization Programme, Ministry of Health, Kinshasa M7MG+2X8, Democratic Republic of the Congo; 2 World Health Organization, Democratic Republic of Congo Country Office, Kinshasa 01206, Democratic Republic of the Congo; 3 Gavi Secretariat, 1218 Le Grand-Saconnex, Switzerland; 4 UNICEF, Democratic Republic of Congo Country Office, Kinshasa M7H9+HQW, Democratic Republic of the Congo; 5 World Health Organization, Regional Office for Africa, Brazzaville P.O. Box 06, Congo

**Keywords:** immunization, vaccine, DR Congo, conference report

## Abstract

Background: At the end of each year, stakeholders of the Essential Immunization Programme (EPI) in the DR Congo meet to review progress made and lessons learned from the implementation of the Annual Operational Plan (AOP) and to set priorities for the following year. This paper presents a conference report that summarizes the main outcomes of the 2025 annual review meeting, which took place from 15 to 20 December 2025, and attracted 76 participants. Conference takeaways: While the 2024 WUENIC data show that the DR Congo is off-track for the 2030 Immunization agenda targets for all antigens, the administrative coverages were reported as optimal in 2025. EPI activities are planned based on administrative coverages, likely overestimated. In 2025, 47% of health zones in North-Kivu, South-Kivu and Ituri (49 out of 104) were fully or partially controlled by armed groups, leading to partial disruptions of immunization service delivery. In 2025, the DR Congo successfully launched the measles–rubella vaccine introduction preceded by a catch-up vaccination campaign in children aged from 6 months to 14 years old and continued to roll out malaria vaccines using a phased approach. Conclusions: Learning from the implementation of the 2025 AOP, the EPI stakeholders adopted a set of priority actions for the immunization programme in 2026.

## 1. Introduction

The Democratic Republic of the Congo (DR Congo) established the Essential Immunization Programme (EPI) in 1978 [1] with the aim of reducing morbidity and mortality due to vaccine-preventable diseases (VPD) in children through life-saving vaccines. The DR Congo is the fourth most-populous country in Africa with a population of 106 million including 4.1 million live births and 3.9 million surviving infants targeted by the EPI [2]. DR Congo is one the five Gavi-eligible high-impact countries that account for 26% of the world’s population and 48% of total birth cohorts in Gavi-eligible countries [3]. The EPI is coordinated through an executive management team at central level and dedicated provincial coordination in each of the country’s 26 provinces. The operational coordination of the EPI is ensured by EPI antennas. There are 51 EPI antennas, an average of two antennas per province. Each antenna oversees an average of 10 health zones, ranging from four to 25.

The DR Congo, through its EPI, started with immunization against six childhood diseases (tuberculosis, poliomyelitis, diphtheria, whooping cough, tetanus and measles) provided to all children aged 0–11 months, and tetanus toxoid vaccination to all pregnant women [4]. Over time, the number of childhood vaccines offered by the EPI was increased to 13 [5]. When the Big Catch-up (BCU) initiative was introduced, the age limit of the target population was extended to 59 months from 2024 to date. The tetanus toxoid vaccine for pregnant women was replaced by the tetanus–diphtheria vaccine.

Since 2002, the implementation of the EPI has been guided and informed by an annual operational plan (AOP) developed jointly by EPI teams from the national and sub-national levels, and technical and financial partners to ensure that all children, in all health zones benefited from life-saving vaccines. The AOP is prepared by a working group set-up by the EPI executive management team and finalized during the annual EPI review meeting, which reviews progress made and lessons learned from the implementation of the EPI during the year and uses this to inform the AOP for the following year. The 2025 annual review took place in Matadi, Kongo Central province, from 15 to 20 December 2025, attracting 76 participants from the 26 provinces and key technical partners such as GAVI, UNICEF, World Health Organization (WHO country officice in DR Congo), the United States Centers for Disease Control and Prevention (US CDC). The meeting included oral presentations on progress made by key components of the 2025 AOP (routine immunization, supplementary immunization activities, introduction of new vaccines, vaccine-preventable disease surveillance and outbreak response, monitoring and evaluation) and by implementing partners. These oral presentations were supplemented by work-group followed by plenary session reports, and a panel of discussions. This paper presents a conference report that summarizes the outcomes of the meeting around three critical areas of specific interest to the DR Congo—immunization coverage, vaccination in fragile settings, new vaccine introduction agenda—and articulates country priorities for 2026, aimed at attaining the national immunization strategy goals in line with Immunization Agenda 2030 (IA2030) [6].

## 2. Conference Report

### 2.1. Immunization Coverage

The 2024 WHO and UNICEF Estimates of National Immunization Coverage (WUENIC) [7] show that the DR Congo is off-track for the IA2030 targets for all antigens: 82% coverage of the first dose of the Diphtheria, Tetanus, Pertussis, *Haemophilus influenzae* type b; Hepatitis B vaccine (DTP) (DTP1), 65% coverage of the third dose of DTP (DTP3) and 55% coverage of the first dose of the Measles-containing vaccine (MCV1). These estimates contrast with the administrative coverages, which were reported as optimal in 2025: 105% for DPT1, 99% for DTP3 and 95% for MCV1.

While WUENIC estimates may reflect the true coverage, EPI activities are planned based on administrative coverages, which are likely overestimated. The use of administrative coverage to monitor the performance of the EPI represents a missed opportunity for intensifying efforts in catching-up vaccination for un- and under-immunized children.

**Figure 1:**
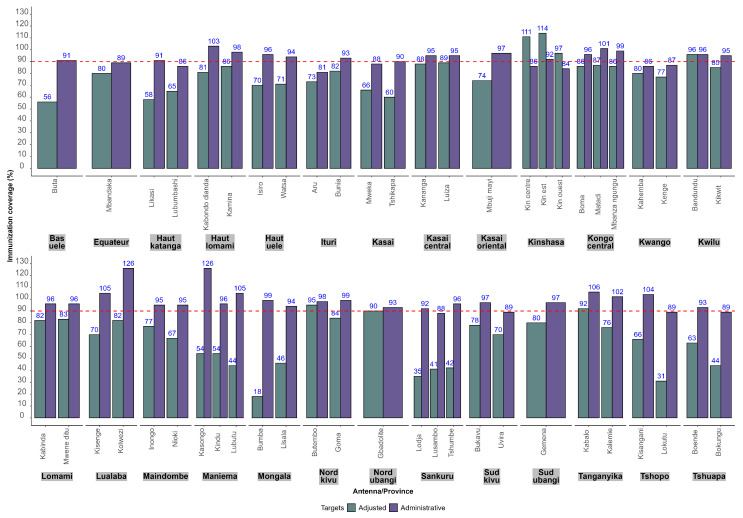
DTP3 coverage by antenna using administrative and adjusted target populations (data from January to September 2025). The red dotted line highlights the target of 90% immunization coverage set for DTP3.

This low coverage may explain the frequent occurrence of measles outbreaks in most health zones. From January to October 2025, 163 health zones out of 519 (31%), in 25 provinces out of 26 (96%) recorded at least one confirmed measles outbreak. The overestimation of administrative coverage is mainly due to underestimates of the target population. In DR Congo the last general population census dates back to 1984 [8]. Every year, the EPI target population is computed by applying the estimated growth rate to the target population of the previous year, leading to possible underestimates. In an attempt to improve estimates of the denominators, the EPI conducted a data triangulation exercise to adjust target population size estimates by health zone using WHO methods for assessing and improving the accuracy of target population estimates for immunization coverage [9]. Figure 1 shows a comparison of immunization coverage with DTP3 by EPI antenna in 2025 as of the end of October, using administrative and adjusted target populations.

In all health zones, administrative immunization coverage with DTP3 was higher than adjusted vaccination coverage, except in the three antennas in Kinshasa Province. A census of the target population was carried out in Kinshasa during the first quarter of 2025 and the results were used as administrative targets. This census was conducted after the target population was adjusted and resulted in a significant increase in the 2025 administrative data compared to 2024. The median increase in the number of surviving infants in 2025 compared to 2024 per health zone in Kinshasa was 38%, ranging from 24% to 129%. The participants recommended that an official guidance note be sent to provinces suggesting the systematic use of adjusted target populations to monitor immunization performance in 2026.

### 2.2. Continuity of Essential Immunization Services in Humanitarian Settings

**Figure 2:**
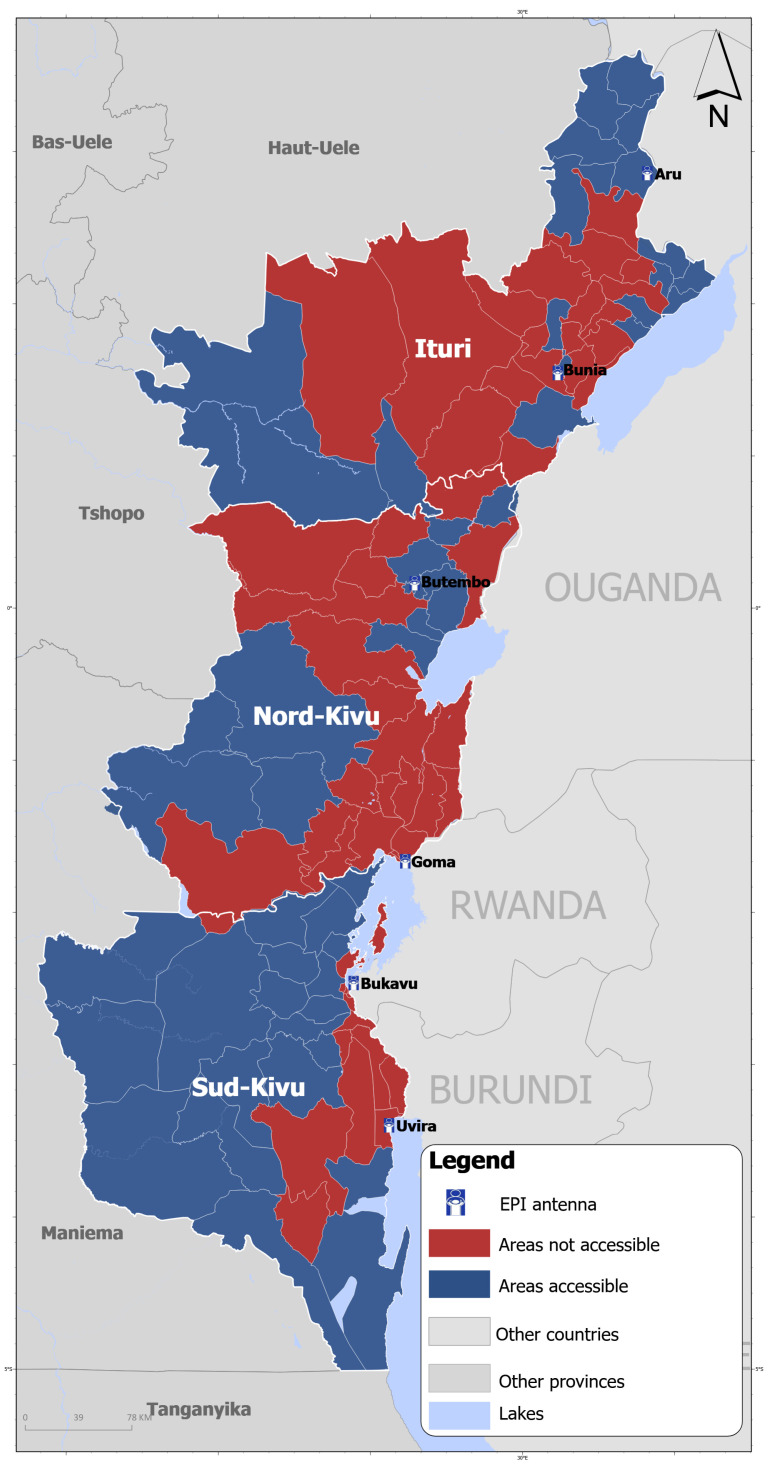
Distribution of health zones by accessibility status due to security risks in Nord Kivu, Sud Kivu and Ituri (data as of 20 December 2025).

Between June and December 2025, 12 health zones out of 34 in North Kivu and 20 out of 34 in South Kivu were located in areas fully controlled by armed groups while 17 health zones out of 36 were partially inaccessible due to security risks in Ituri [10,11]. In total, there was limited access by national authorities to 49 health zones out of 104 (47%) (Figure 2). This led to partial disruptions in vaccine distribution, immunization service provision, and vaccine demand in territories partially or fully outside the control of national authorities in 2025. The two EPI antennas of South Kivu (Bukavu, Uvira) and one of the two antennas of North Kivu (Goma) are based in areas outside national authorities’ control. As per the International Committee of the Red Cross [12], staff shortage affected 40% of health facilities in North and South Kivu provinces, while 13% were completely nonfunctional. Despite this context, a certain level of continuity of immunization services has been maintained in areas not fully controlled by national authorities, evidenced by the continuation of vaccine distribution, the levels of completeness in reporting routine immunization data and immunization coverages.

The Goma and Bukavu antennas are supplied with vaccines from the Butembo antenna, while the Uvira antenna is supplied from Kalemie (Tanganyika province), with UNICEF support. The distribution of vaccines to health zones in territories not controlled by national authorities is usually done in two ways: (i) vaccines collected by the health zones that are equipped with vehicles, (ii) assistance from humanitarian organizations such as Atlas Logistic, the United Nations High Commissioner for Refugees, and Doctors without Borders. From January to September 2025, the completeness of monthly reports of routine immunization data in the second version of the District Health Information Software (DHIS2) version 42.4.0 in areas with security issues or occupied by armed groups was 86% in Ituri (versus 88% in areas controlled by the Government), 91% in North Kivu (versus 88% in areas controlled by the Government) and 86% in South Kivu (versus 95% in areas controlled by the Government). The median coverage with DTP3 in areas with security issues or occupied by armed groups was 94% [range: 56%; 110%] in Ituri, 102% [range: 71%; 150%] in North Kivu and 112% [range: 87%;164%] in South Kivu. The number of health zones with less than 80% DTP3 coverage in areas with security issues or occupied by armed groups was four out of 17 in Ituri (23%), two out of 12 in North Kivu (17%) and none out of 20 in South Kivu. These results showed that the disruptions of routine immunization in humanitarian settings in Ituri, North Kivu and South Kivu were limited to a few health zones, as a result of insufficient supervision of immunization points of delivery, limited data quality control/assessment, and reduced funding for outreach and mobile vaccination sessions. Coverage above 100% occurs mainly in health districts that hosted internally displaced people and recorded returnees. These results highlight the need for ensuring that supportive supervision and data quality assurance activities continue to be carried out in areas partially or fully controlled by armed groups. It is critical, in armed conflict settings, that immunization service delivery is included in the Health Resources and Services Availability Monitoring System (HeRAMS) [12], so that immunization service gaps are identified and addressed as part of the public health humanitarian response.

### 2.3. Advancing the New Vaccine Introduction Agenda

In 2025, the DR Congo planned to continue implementing the phased approach of malaria vaccine introduction and to launch the introduction of the measles–rubella (MR) combined vaccine.

#### 2.3.1. Update on Malaria Vaccine Introduction

Malaria remains the first cause of morbi-mortality in DR Congo among children under five years old [13]. Following WHO prequalification of two vaccines (RTS/AS01 approved in 2022 [14] and R21 approved in 2023 [15]), the DR Congo added the R21 malaria vaccine to its malaria control interventions package in 2024. The country received support from Gavi for the acquisition and deployment of the vaccines in 441 health zones with moderate and high transmission rates, as per WHO recommendations. The R21 vaccine schedule is four doses including three primary doses to be administered at 6, 7, and 8 months, followed by a booster dose at 15 months.

The DR Congo started to roll out the malaria vaccine in October 2024 in one of the 26 provinces (Kongo central). The introduction was extended to eight additional provinces as part of the first block (Kwilu, Kwango, Mai-Ndombe, Équateur, Mongala, Nord-Ubangi, Sud-Ubangi and Tshuapa) in May and June 2025, and in the second block of seven provinces (Maniema, Haut Lomami, Lomami, Kasaï central, Haut Uele, Bas Uele, Tshopo) in August 2025.

As of the end of October 2025, the administrative coverage in the 16 provinces that have rolled out malaria vaccine was 86% with the first dose (target: 70%), 78% with the second dose (target: 62%) and 60% with the third dose (target: 60%). These results, which seem to be optimal, may be overestimated given the underestimation of target populations. The post-introduction evaluation and the national immunization coverage survey scheduled in 2026 will provide a better estimation of malaria vaccine coverage.

Introduction in the 10 remaining provinces has been delayed and is scheduled for 2026. The 78 health zones with low transmission of malaria, not eligible for Gavi support, are located in these provinces. Introduction in these provinces may have ethical and political implications as people in health zones with low transmission may not understand why the vaccines are available in some health zones but not in theirs. The Ministry of health is exploring ways of raising local resources through public–private partnerships to cover the very high cost of vaccines (US$ 3.99 per vaccine dose, i.e., US$ 11.5 million to cover 70% of the target population in the 78 health zones allowing 995 737 children to be reached in the first year of introduction).

#### 2.3.2. Measles–Rubella Vaccine Introduction

Since 2010, the DR Congo has faced recurring measles outbreaks affecting all 26 provinces. Among samples from suspected measles cases that tested negative for measles, more than 10% tested positive for rubella. While rubella is generally mild, it can cause congenital rubella syndrome, a major cause of congenital anomalies [16].

To put the country on track towards achieving measles elimination and reduce the burden of congenital rubella syndrome, the DR Congo decided to replace the measles-containing vaccine (MCV) with the combined MR vaccine in the national immunization schedule, starting in 2025. To close immunity gaps among children and adolescents for measles and rubella, the country agreed to carry out catch-up vaccination campaigns targeting around 62 million children aged from 6 months to 14 years.

The first of the three blocks of phased MR introduction and catch-up vaccination took place from 27 November to 1 December 2025. The percentage of target population reached in the seven provinces from the first block was 96.7%. Four provinces out of seven surpassed the 95% coverage target set by the EPI, except Haut Katanga (91.5%), Ituri (92.2%) and Lualaba (93.7%). In Lualaba and Haut-Katanga, some health zones delayed the campaign launch due to disputes over unpaid service fees from previous vaccination campaigns. In Ituri, 47% of health zones were partially inaccessible for security reasons, disrupting the campaign. The delay in funding operational costs at the lowest level (health zones), the late distribution of AEFI (adverse events following immunization) treatment kits, the delay in starting demand-generation activities, in addition to unpaid service fees from previous campaigns were the main challenges faced during the first block.

The second block (11 provinces) and the third block (8 provinces) are scheduled for March and May 2026, respectively, and will be integrated with polio national immunization days.

## 3. Conclusions and Priority Actions for 2026

Learning from the implementation of the 2025 plan, the EPI and partners adopted the following set of priority actions for the immunization programme in 2026:

• Keep the momentum gained through implementation of the Mashako Plan, which aims to revive routine immunization in the DR Congo [5] by ensuring continuity of immunization service delivery at all levels of the health systems. This is despite a significant reduction in funding from Gavi, the leading financial partner of the immunization programme in the DR Congo. The Mashako Plan was set up by the Ministry of health in 2018 [5] to improve the performance of routine immunization and decrease childhood mortality from VPD, using the reaching-every-district approach [17].
• Carry out post-introduction evaluation in the 16 provinces that have started to roll out malaria vaccines.
• Extend the malaria vaccine rollout to the third block of 10 provinces, starting with provinces that have fewer than five health zones of low transmission, while exploring ways of mobilizing funds for the deployment of vaccines in all low-transmission health zones.
• Continue introducing the combined MR vaccine, preceded by the catch-up vaccination campaign targeting children and adolescents aged from 6 months to 14 years, in blocks 2 (11 provinces) and 3 (8 provinces).
• Continue to implement the plan for improving routine immunization data quality to close the gap in immunization coverage between administrative data and surveys.
• Conduct a national immunization coverage survey in 2026 since the last one dates back to 2024, covering the 2023 cohort.

The annual review offered the opportunity to all immunization programme stakeholders in DR Congo to review progress made in 2025, draw on lessons learned and build a consensus on the main priorities in 2026. Such meetings are critical for the continuous improvement of immunization programme performance and for strengthening the commitment of all stakeholders towards common objectives. The EPI in DR Congo is implemented in a complex context, including disruption of immunization services in fragile settings, underestimation of target population preventing timely decision making, strong dependence on funding from external partners, weak health systems and suboptimal integration of EPI with other health programmes.

### Limitations

This paper is a meeting report. The content was developed using oral presentations and summaries of work groups and panel discussions and was not based on a specific study conducted by the authors. No prior independent verification was conducted. Malaria vaccine introduction and Measles–Rubella catch-up vaccination results were based on health facility reports and administrative targets. No immunization coverage survey has been conducted in 2025. The use of administrative targets, definitely underestimated, may be misleading. The interpretation of the results presented here should take this limitation into account.

## Data Availability

All the presentations and materials from the 2025 EPI annual review of the essential programme on immunization in DR Congo are available and downloadable on the following link: https://worldhealthorg.shinyapps.io/matadi2025/ (accessed on 8 March 2026).
